# Untangling the relationships among regional occupancy, species traits, and niche characteristics in stream invertebrates

**DOI:** 10.1002/ece3.1076

**Published:** 2014-04-19

**Authors:** Jani Heino, Mira Grönroos

**Affiliations:** 1 Finnish Environment Institute, Natural Environment CentreP.O. Box 413, FI-90014, Oulu, Finland; 2 Department of Biology, University of OuluP.O. Box 3000, FI-90014, Oulu, Finland

**Keywords:** Body size, invertebrates, niche breadth, niche position, species traits, streams

## Abstract

The regional occupancy and local abundance of species are affected by various species traits, but their relative effects are poorly understood. We studied the relationships between species traits and occupancy (i.e., proportion of sites occupied) or abundance (i.e., mean local abundance at occupied sites) of stream invertebrates using small-grained data (i.e., local stream sites) across a large spatial extent (i.e., three drainage basins). We found a significant, yet rather weak, linear relationship between occupancy and abundance. However, occupancy was strongly related to niche position (NP), but it showed a weaker relationship with niche breadth (NB). Abundance was at best weakly related to these explanatory niche-based variables. Biological traits, including feeding modes, habit traits, dispersal modes and body size classes, were generally less important in accounting for variation in occupancy and abundance. Our findings showed that the regional occupancy of stream invertebrate species is mostly related to niche characteristics, in particular, NP. However, the effects of NB on occupancy were affected by the measure itself. We conclude that niche characteristics determine the regional occupancy of species at relatively large spatial extents, suggesting that species distributions are determined by environmental variation among sites.

## Introduction

A positive relationship between regional occupancy and local abundance is among the most general ecological patterns (Hanski 1982; Brown 1984; Gaston and Blackburn 2000; Blackburn et al. 2006). Such a relationship occurs in a wide range of organismal groups, ranging from algae (e.g., Soininen and Heino 2005) and bryophytes (e.g., Heino and Virtanen 2006) to birds (e.g., Gaston et al. 1998) and mammals (e.g., Blackburn et al. 1997). Despite the fact that a large number of studies have reported strongly positive occupancy–abundance relationships, relatively few studies have directly examined potential factors underlying the relationship (Gaston et al. 1997; Gregory and Gaston 2000; Brändle and Brandl 2001; Tales et al. 2004) or have assessed possible differences among biologically defined groups of species (Quinn et al. 1997; Cowley et al. 2001; Holt and Gaston 2003; Foggo et al. 2007). These biological groupings may be broad trophic guilds (e.g., Heino 2008), dispersal modes (e.g., Foggo et al. 2007), body sizes (e.g., Tales et al. 2004), and groups of species using similar habitats (e.g., Holt and Gaston 2003). These types of studies have thus examined variation in occupancy and abundance in relation to various species traits. We examined four fundamental biological trait groups (i.e., feeding guild, habit trait, dispersal mode, body size) and two niche measures (i.e., niche breadth [NB], niche position [NP]) as correlates of occupancy and abundance in stream invertebrates. We considered the occupancy–abundance relationship in stream invertebrates and then proceeded to examining variation among species in occupancy or mean local abundance in relation to various biological and ecological traits.

First, if one considers feeding guilds, which include species utilizing the same resources in a similar way (Hawkins and MacMahon 1989; Fauth et al. 1996), among-group differences in occupancy and abundance may stem from the fact that resource distribution and abundance for different groups varies in the system studied. For example, one could assume that predators are strongly limited by prey abundance and thus the abundance of predatory species is lower than that of nonpredatory species due to their position in the food chain (Elton 1927; Gaston and Kunin 1997). Furthermore, if there is a strong relationship between the occupancy and abundance of species, species in trophic groups exhibiting lower local abundance should also show more restricted regional distributions (Gaston et al. 2000; Heino 2008). These ideas remain to be tested rigorously in both terrestrial and aquatic systems. In stream systems, invertebrates are divided among a number of feeding guilds (i.e., functional feeding groups; Cummins 1973; Cummins and Klug 1979). Six feeding guilds, including filterers, gatherers, predators, parasites, scrapers and shredders, were examined here, with each of these groups having different trophic roles in streams (Merritt and Cummins 1996; Wallace and Webster 1996). It can be hypothesized that the feeding guild of a species also mirrors the distribution and abundance of food resources in stream systems, with predator and parasite species being less abundant locally than species in the other feeding guilds (see also Statzner et al. 2008).

Second, habitat use may also affect occupancy and abundance, with species utilizing common microhabitats being more widely distributed and locally more abundant than those utilizing rare microhabitats (Venier and Fahrig 1996). This hypothesis can also be related to biological characteristics of species. For example, in stream systems, invertebrates are divided among habit traits (Merritt and Cummins 1996), which describe the main types of habitat use of species. Of these habit traits, burrowers, climbers, clingers, sprawlers and swimmers are the most commonly represented in northern headwater streams (Heino 2005b). Based on a limited amount of evidence, it appears that swimmers and clingers are both regionally widely distributed and locally common, whereas burrowers and climbers tend to show more limited degrees of occupancy and abundance in headwater streams (Heino 2005b). These differences among habit trait groups (HTGs) may be related to the ecological success of swimmers (e.g., they can easily enter drift dispersal) and clingers (e.g., they can resist stream currents by clinging on stones by claws and other morphological structures) in stream riffle sites (see also Merritt and Cummins 1996). These two HTGs thus represent contrasting, yet successful adaptations to living at stream riffle sites.

Third, dispersal mode is a major species trait affecting occupancy and abundance (Foggo et al. 2007; Verberk et al. 2010). It can be predicted that strongly actively dispersing species have wider regional distributions than those with weaker passive dispersal ability. Species with stronger active dispersal ability may either be locally more abundant, on average, if rescue effects sustain populations at sink sites (Pulliam 1988) or locally scarcer if emigration from source sites reduces mean population size across sites (Verberk et al. 2010). Although these predictions are difficult to test directly, examining differences in occupancy and abundance between dispersal mode groups (DMGs) may shed light into that issue. Differences in occupancy and abundance were examined here among three DMGs of stream invertebrates, including passive aquatic, passive terrestrial and active terrestrial species (Grönroos et al. 2013; Heino 2013). It can be hypothesized that active terrestrial species are more widely distributed and locally more abundant than passive terrestrial and passive aquatic species (see also Bilton et al. 2001). This is because active terrestrial species should be more able to track variation in environmental conditions among sites, allowing species to find suitable sites across a region and be more widely distributed than passive species (Heino 2013).

Fourth, body size (BS) has been shown to be related to the distributions of species. Although some work has suggested that very small species are almost ubiquitously distributed globally (Finlay 2002; Finlay and Fenchel 2004), the degree to which this suggestion holds for stream invertebrates, which show relatively little variation in BS in comparison with that existing in the whole biota, is not known (Passy 2012). For example, macroscopic invertebrates in boreal streams range in size from a millimeter up to six centimeters, allowing ecologists to examine the effects of BS on occupancy and abundance in relatively similar-sized organisms. It can be hypothesized that small invertebrate species are locally more abundant than larger species (Statzner et al. 2008), but the degree to which regional occupancy varies with BS in stream invertebrates is difficult to judge. It can either be that small species are carried long distances passively or that large species are more able to fly long distances, which may be seen in the degree of occupancy (Passy 2012).

In addition to biological traits, two important ecological characteristics affecting the occupancy and abundance of a species are its niche position (NP) and niche breadth (NB) (Gaston et al. 2000; Tales et al. 2004; Heino 2005a). There is a large body of theory on the relationships among occupancy, abundance and niche characteristics (Brown 1984; Hanski et al. 1993; Passy 2012). Niche-based models predict, for example, that both occupancy and abundance mirror the degree to which local environmental conditions meet the requirements of species (Brown 1984; Slatyer et al. 2013). While Brown's (1984) hypothesis is mainly related to NB (i.e., the broader the niche, the wider the regional occupancy and the higher the local abundance), a hypothesis related to NP may also explain variation in occupancy and abundance among species (Hanski et al. 1993; Venier and Fahrig 1996). This hypothesis predicts that species having marginal niches (i.e., low habitat availability and a high degree of marginality in environmental preferences) are less widely distributed and locally less common than species capable to occur in average habitat conditions (i.e., high habitat availability and a low degree of marginality in environmental preferences). Support for the niche-based models, although rather weak, has been found previously for stream invertebrates at the drainage basin scale (Heino 2005a; Siqueira et al. 2009), but next to nothing is known if the relationship holds at the across-drainage basins scale where environmental gradients are much larger than at the within-drainage basin scale (Passy 2012). It can be hypothesized that NP and NB are much more important for occupancy at regional extents than at very small local or very large continental scales. This is because species environmental niches may be more important at regional scales, whereas historical factors may mask their influences at continental scales (Brändle and Brandl 2001). Furthermore, although many studies have pointed to the importance of NB, few studies have actually simultaneously compared NB and NP as correlates of occupancy (Slatyer et al. 2013).

These conjectures were tested based on surveys of stream invertebrates across three northern drainage basins. We did not coin formal hypotheses for the relationships between biological traits and occupancy or abundance, as these relationships are still unclear and previously little reported. By contrast, there are many theoretical and empirical findings on the relationships between niche characteristics and occupancy or abundance. Thus, we expected that occupancy and abundance should be positively related to NB (Passy 2012; Slatyer et al. 2013) and negatively related to NP (i.e., niche marginality; Gregory and Gaston 2000; Tales et al. 2004; Heino 2005a). Finally, we also expected that the full model of all traits that explain variation in occupancy should be ruled by variables such as local abundance, NP and NB due to their profound influence on the occupancy of species (Hanski et al. 1993; Venier and Fahrig 1996; Gaston et al. 2000; Blackburn et al. 2006).

## Materials and Methods

### Study areas and stream types

The test data set comprised surveys of macroinvertebrates in three northern drainage basins. Partly the same data set as used in the present study has been formerly utilized in examining community–environment relationships and metacommunity patterns in stream macroinvertebrates (Heino et al. 2012; Heino 2013; Schmera et al. 2013; Grönroos et al. 2013). These studies found clear variation in multiple traits at the community level along environmental gradients and virtually no spatial structuring of taxonomic community composition within each drainage basin. The following description of the drainage basins and field methods is largely based on two previous studies (Heino 2013; Schmera et al. 2013). The details of the study areas will, however, be reiterated here to facilitate understanding the ecological context of the three drainage basins. Altogether 60 near-pristine to pristine streams were included in the present study, and they covered latitudes 65°N to 70°N and longitudes 26°E to 30°E.

The first study area is located in the Iijoki drainage basin (centered on 65°N, 27°E). The study area is characterized by boreal coniferous forests and peatlands. The streams are generally slightly acidic, and nutrients range from low to moderate (Heino et al.*,* 2012; Heino 2013). A total of 20 first- to third-order streams were surveyed in the Iijoki drainage basin. A second study area is located in the Koutajoki drainage basin in northeastern Finland (centered on 66°N, 29°E). Headwater streams in the drainage basin are characterized by circumneutral to alkaline water, low to high levels of humic substances, and low-to-moderate nutrient concentrations (Heino et al., 2012; Heino 2013). A total of 20 first to third-order streams were sampled in the Koutajoki drainage basin. A third study area is located in the Tenojoki drainage basin (centered on 70°N, 27°E). This subarctic study area is characterized by arctic-alpine vegetation, comprising mountain birch woodlands at low altitude and barren tundra at higher altitude. Stream waters are circumneutral, and nutrient levels are indicative of highly oligotrophic systems (Heino 2013; Schmera et al. 2013). Of the 30 streams sampled in this drainage basin, 20 streams were randomly selected for the present study to guarantee that sample sizes were the same for each drainage basin. The data from the three drainage basins were combined for the analyses, as previous occupancy–abundance studies of stream invertebrates have largely neglected across-drainage basins phenomena and instead concentrated on within-drainage basin patterns (Heino 2005a,b2005b; Siqueira et al. 2009).

### Environmental variables

Several riparian, in-stream habitat and water chemistry variables were measured at each site (Heino et al., 2012; Heino 2013). Cover (%) of deciduous trees was assessed in a 50-meter section on both banks directly upstream of the sampling site. Shading was estimated visually as percent canopy cover at the whole study section. Current velocity (at 0.6 × depth) and depth were measured at 30 random locations along cross-stream transects, the number of which depended on stream width. Mean wetted width of each stream was measured based on five cross-stream transects. Macrophyte cover (%) and substratum particle class cover (%) were assessed at 10 random randomly spaced 50 × 50 cm plots. In addition, in each of the 10 plots, visual estimates of the percentage cover of five particle size classes were made based on a modified Wentworth scale: (i) sand (diameter 0.25–2 mm), (ii) gravel (2–16 mm), (iii) pebble (16–64 mm), (iv) cobble (64–256 mm), and (v) boulder (256–1024 mm). Standard deviations of velocity, depth, and macrophyte cover were also used as variables describing habitat heterogeneity at each site. Water samples were collected simultaneously with the field sampling or determined in the field, and they were analyzed for pH and conductivity (National Board of Water and the Environment 1981). For variation in the environmental variables measured, see Heino (2013) and Schmera et al. (2013). These previous studies have shown that the three drainage basins studied differ in environmental conditions, thus widening the environmental gradients to a considerable degree in comparison with studies conducted within a single drainage basin.

### Macroinvertebrate sampling

Stream macroinvertebrates were sampled in the Koutajoki drainage basin in the last week of May in 2008, in the Iijoki drainage basin in the last week of May in 2009, and in the Tenojoki drainage basin in the second week of June in 2010. As the resources for this study did not allow sampling all the sites across the three basins within a short period of time in a single year, the sites were sampled in different basins in different years (Heino 2014). It is actually more important to sample the sites in the same season (e.g., soon after the snowmelt in the spring) than in the same year. If the sites are not sampled within a short period of time in the same season, the results may not mirror spatial differences but instead show seasonal differences in stream macroinvertebrate communities (Schmera et al. 2013). Spring after the snowmelt is also the season when the majority of macroinvertebrates in high latitude streams are still in the larval stage. The timing of sampling also facilitated the identification of aquatic insect larvae, most of which are close to their maximum size at this time of the year (Heino et al. 2009).

At each site, the field crew took a collective 2-min kicknet (net mesh size 0.3 mm) sample covering most microhabitats present in a riffle site (Heino et al., 2012; Grönroos et al. 2013). This sampling effort typically yields a majority of species occurring at a site in a given season, mainly missing rare species that occur only sporadically in streams (Mykrä et al. 2006). Such a sample generally yields hundreds to thousands of macroinvertebrate individuals in northern streams. Macroinvertebrates and associated material were immediately preserved in ethanol (70%) in the field, and samples were taken to the laboratory for further processing and identification. Macroinvertebrates were identified to species, species group, or genus.

### Species traits: biological groupings of stream invertebrates

Macroinvertebrates were assigned into six feeding guilds according to Merritt and Cummins (1996), Moog (2002), and Vieira et al. (2006). The feeding guilds included shredders, gatherers (=detritivores), filterers, scrapers (=grazers), predators, and parasites. Some classifications take flexibility in the feeding modes into account by a point scoring system (Schmedtje and Colling 1996; Moog 2002), where scores are assigned to each taxa with regard to functional feeding groups it represents (e.g., six points for scraper and four points for gatherer of the total of ten points). Thus, in this example, a species would be assigned to the scraper guild. In general, a species with ≥5 points for a given functional feeding group was assigned to belong to the respective group (Heino et al. 2009; Grönroos and Heino 2012). As we had to assign each species into a single feeding guild only (i.e., “feeding guild” was a categorical variable in the analyses), we could not use the more defined point scoring system.

Macroinvertebrates were also assigned into habit traits according to Merritt and Cummins (1996). Although this reference is concerned with North American taxa, the same genera occur in our northern Finnish study area. There were five habit traits in the present data, including burrowers, clingers, climbers, sprawlers, and swimmers. These habit traits are generally dominating in the riffle sites of boreal and subarctic headwater streams (Heino 2005b).

Four size classes of maximum larval body lengths of macroinvertebrates were also used in this study, based on information provided by personal communication with Sylvain Dolédec (most groups), Jari Ilmonen (Simuliidae), and Lauri Paasivirta (Chironomidae). The four size classes used were the following: 0–5 mm, 5–10 mm, 10–20 mm, and 20–40 mm. In general, larval body length correlates with that of adult body length, although due to morphological variation among invertebrate taxa, that correlation is not perfect.

Macroinvertebrates were also divided into three DMGs (Grönroos et al. 2013; Heino 2013). These groups were (i) aquatic passive dispersers (DM1), containing species with no winged adult stage; (ii) aerial passive dispersers (DM2), containing the dipteran family Chironomidae; and (iii) active aerial dispersers (DM3), containing other insect families. Although this division is coarse, more detailed information on the dispersal modes or abilities is not available for most of the taxa found in the study region.

Although more sophisticated trait and life history variables would certainly be helpful in this type of studies (see Verberk et al. 2013), we had to rely on the above-mentioned trait information, as virtually no other data are currently available for the species studied. This is especially true for non-biting midges (Diptera: Chironomidae), which comprised a large proportion of species detected in our study sites.

### Data analysis

Niche position and NB were measured using the outlying mean index (OMI) analysis (Dolédec et al. 2000). This method measures the marginality of species habitat distribution, that is, the distance between the mean habitat conditions used by a species and the mean habitat conditions in the study area. The position of a species depends on its deviation from the distribution of a hypothetical species that tolerates “average” habitat conditions and is uniformly distributed across all habitat conditions. The OMI index thus measures the NP of a species, and species having high values of OMI have marginal niches (i.e., low habitat availability), and those that have low values have non-marginal niches (i.e., high habitat availability). A variance term based on this method is species tolerance that measures the range in the distribution of a species along the sampled environmental gradients or, in more general terms, its NB. Species that have high values of tolerance occur across broad environmental ranges (i.e., wide habitat NB), and those that get low values occur only across a limited range of conditions (i.e., small habitat NB) (Tales et al. 2004; Heino 2005a). NP (OMI) and NB (species tolerance) were computed for each species using the OMI analysis in the R package *ade4* (Chessel et al. 2012). The analysis was based on log-transformed species abundance data and the 15 environmental variables measured in this study. These variables thus defined the realized habitat niches for each species with regard to the measured environmental variables.

To determine the utility of species tolerance as a measure of NB, Levins' (1968) measure of NB (LNB), which is independent of environmental variation, was also calculated for each species. LNB is a function of the uniformity of distribution of species abundance among “the resource states” or the sites surveyed (Pandit et al. 2009; Devictor et al. 2010). LNBs were calculated for each species using the R package *spaa* (Zhang 2013).

Prior to the analyses described later, distributions of continuous variables were checked based on Shapiro–Wilk normality test and normality plots. For general linear models, the proportion of sites occupied was logit-transformed, and mean local abundance at occupied sites, NP and NB was log-transformed to better meet the assumptions of parametric tests. These transformations of occupancy and mean local abundance are typically used in studies examining interspecific variation in occupancy or abundance using general linear models (Verberk et al. 2010).

General linear models were used to analyze variation in the proportion of sites occupied or mean local abundance (based on occupied sites only). Four models were analyzed: (i) Occupancy ∼ NP + NB + FFG + HTG + DMG + BS; (ii) Mean abundance ∼ NP + NB + FFG + HTG + DMG + BS. We also used Akaike information criterion (AIC; Burnham and Anderson 2002) in the context of forward-backward selection of explanatory variables to select the best reduced models. Thus, we also reported (iii) a reduced model explaining occupancy and (iv) a reduced model explaining mean local abundance. In all models, variance inflation factors (VIF) were typically clearly below 10, a threshold often considered important in evidencing severe collinearity among explanatory variables (Kutner et al. 2004).

Furthermore, linear regression analysis was run to examine the relationships among the continuous variables, and Kruskal–Wallis test was used to test for significant differences in the proportion of sites occupied or mean local abundance at occupied sites among the categorical variables. Exploratory analyses, general linear models and Kruskal–Wallis tests were run using the R package *Rcmdr* (Fox 2005). Furthermore, variation partitioning based on partial linear regression analysis was employed to estimate the pure and shared proportions of variation in occupancy among NP, NB, and mean local abundance. Variation partitioning was run using the R package *vegan* (Oksanen et al. 2013).

No correction for phylogeny was used in the above-mentioned analysis, as none is currently available for the species included in the data. However, as a proxy for phylogeny in the present study, we used the categorical variable “taxonomic order” and tested for significant differences among orders in occupancy, mean local abundance, NP, and NB. We did not find any indication that taxonomy accounted for significant variation in these variables (Kruskal–Wallis test, all *P* > 0.210). Additionally, previous studies have found that the occupancy–abundance relationship remains largely unchanged had a phylogeny correction been conducted or not (Blackburn et al. 1997; Cowley et al. 2001; Holt and Gaston 2003; Tales et al. 2004). Furthermore, a previous study on stream insects in a boreal drainage basin showed that congeneric and confamilial species varied widely in the degree of occupancy and mean local abundance, suggesting that phylogenetic relationships among species did not affect the results (Heino 2005a).

## Results

**Figure 1:**
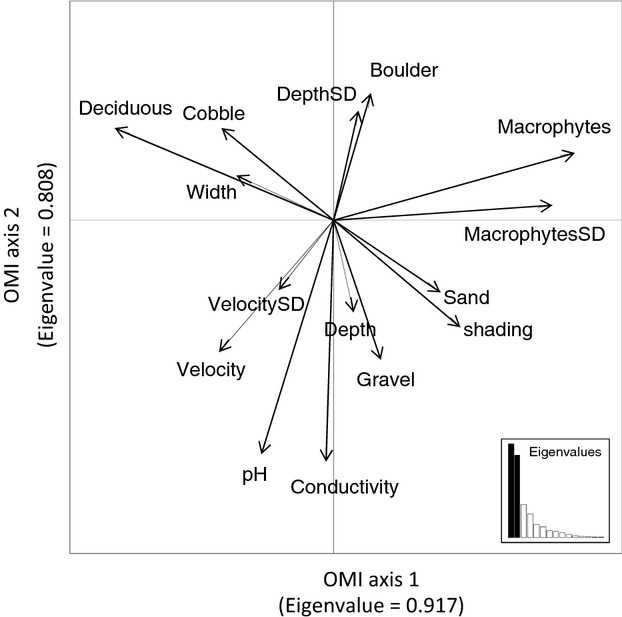
Canonical weights of the 15 environmental variables which defined the niche parameters of species in the outlying mean index (OMI) analysis. The length of an arrow describes the relative importance of each variable in the analysis, and the direction of the arrow indicates among-variable correlations.

Of the total of 226 species detected in the samples from the three drainage basins, data for 126 species occurring at least at three sites were used in the analyses below, because NP and NB could be reliably calculated for only this subset of species. The OMI analysis showed that macrophyte cover (OMI axis 1), riparian deciduous tree cover (OMI axis 1), conductivity (OMI axis 2), and pH (OMI axis 2) were the most important environmental variables related to the distributions of species across the 60 streams (Fig. 1). There were clearly marginal and nonmarginal species, with high and low OMI index values (i.e., NP), respectively. Similarly, clear specialist and generalist species were detected, with low and high values of tolerance (i.e., NB), respectively.

**Figure 2:**
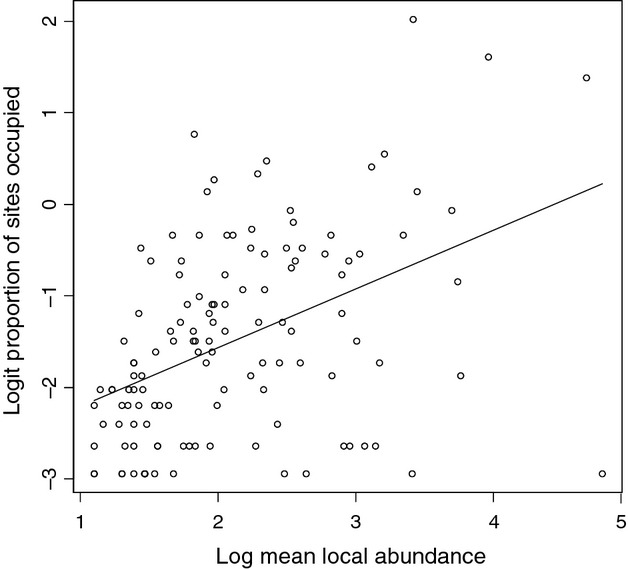
The relationship between occupancy and mean local abundance at occupied sites in stream invertebrates. Model statistics: adjusted *R*^2^ = 0.190, *F* = 30.340, *P* < 0.001.

**Figure 3:**
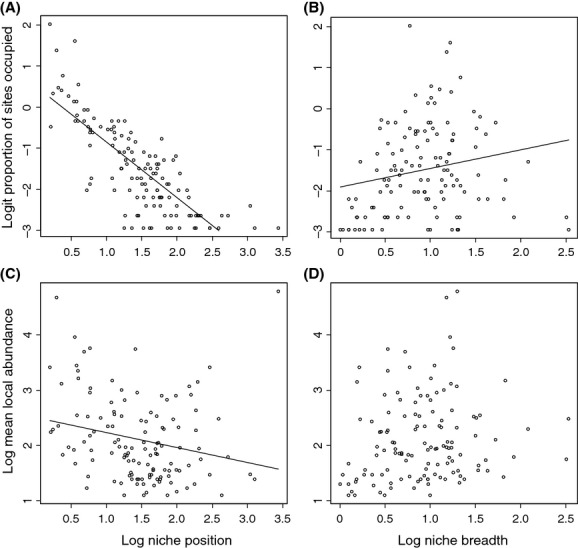
Relationships between (A) occupancy and niche position (NP); (B) occupancy and niche breadth; (C) mean local abundance at occupied sites and NP; and (D) mean local abundance at occupied sites and niche breadth. N = 126 species. NP and niche breadth were based on the outlying mean index (OMI) analysis.

**Figure 4:**
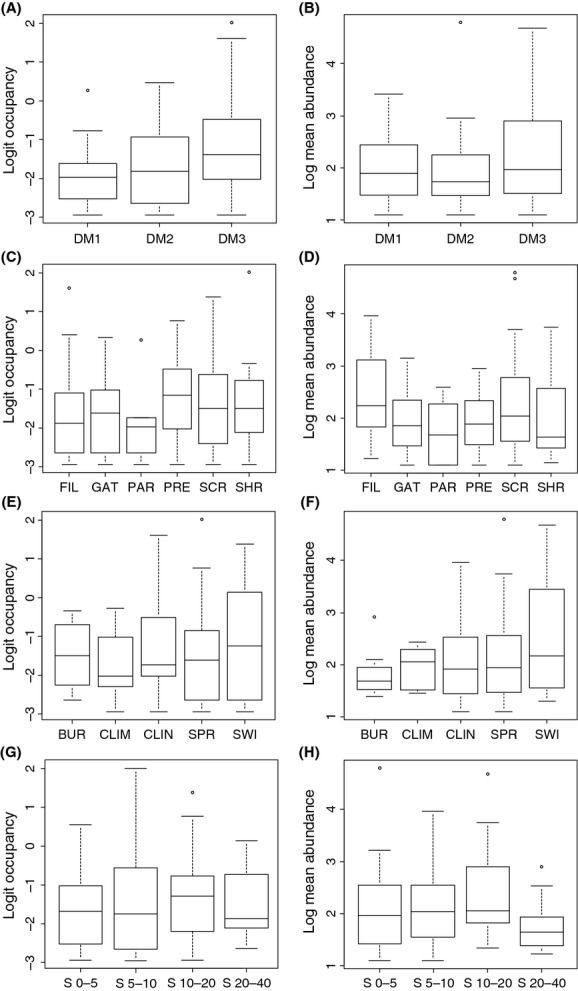
Box plots of variation in occupancy and mean local abundance at occupied sites among different biological groups of species. Figures portray variation among (A, B) dispersal mode groups, (C, D) feeding guilds, (E, F) habit traits, and (G, H) body size classes. See main text for abbreviations.

As expected, occupancy was positively related to mean local abundance, although the relationship was not particularly strong (adj. *R*^2^ = 0.190, *F* = 30.340, *P* < 0.001; Fig. 2). NP was strongly negatively related to occupancy (adj. *R*^2^ = 0.626, *F* = 211.100, *P* < 0.001), whereas it only accounted for a minor amount of variation in mean local abundance (adj. *R*^2^ = 0.044, *F* = 6.821, *P* = 0.010) (Fig. 3A and C). In contrast, NB explained poorly variation in occupancy (adj. *R*^2^ = 0.032, *F* = 5.169, *P* = 0.024), and it was not significantly related to mean local abundance (Fig. 3B and D). Of the biological traits of species, there were significant differences among the DMGs in occupancy (Kruskal–Wallis test *χ*^2^ = 6.397, *P* = 0.040), with active terrestrial dispersers showing higher occupancy than passive species. Occupancy did not differ significantly among feeding guilds, among habit traits or among BS classes. There were no significant differences in mean local abundance among the trait groupings of species (Fig. 4).

The general linear model explaining variation in occupancy showed that only NB and NP were significant predictors of occupancy, whereas the other traits were not significantly related to occupancy (Table 1A). The reduced model thus included only NP and NB, and this model explained a bit more variation in occupancy than the full model (Table 1B). In contrast, variation in mean local abundance was not significantly related to NB, but instead NP and dispersal mode accounted for some significant variation in mean local abundance (Table 2A). The reduced model included NP, dispersal mode, functional feeding mode and BS, and this model had basically the same explanatory power as the full model (Table 2B).

**Table 1:** General linear models explaining variation in the occupancy of species (logit-transformed proportion of sites occupied)

	Sum square	df	*F* value	*P*
(A)				
logNP	84.686	1	200.244	<0.001
logNB	3.600	1	8.512	<0.001
DMG	0.059	2	0.069	0.932
FFG	1.225	5	0.579	0.715
HTG	1.685	4	0.996	0.412
Size	0.558	3	0.439	0.724
Residuals	46.0.98	109		
(B)				
logNP	93.129	1	225.085	<0.001
logNB	4.629	1	11.188	0.001
Residuals	50.891	123		

(A) Full model statistics: adjusted *R*^2^ = 0.647, *F* = 15.360, *P* < 0.001. (B) Reduced model statistics: adj. *R*^2^ = 0.655, *F* = 119.800, *P* < 0.001. Reduced model was based on AIC. AIC, Akaike information criterion; NP, niche position; NB, niche breadth; DMG, dispersal mode group; FFG, functional feeding guild; HTG, habit trait group.

**Table 2:** General linear models explaining variation in the log-transformed mean local abundance of species

	Sum square	df	*F* value	*P*
(A)				
logNP	2.151	1	4.370	0.038
logNB	0.956	1	1.943	0.166
DMG	4.454	2	4.525	0.012
FFG	4.175	5	1.696	0.141
HTG	1.752	4	0.890	0.472
Size	2.407	3	1.630	0.186
Residuals	53.638	109		
(B)				
logNP	2.185	1	4.433	0.037
DMG	4.106	2	4.416	0.017
FFG	4.722	5	1.1916	0.097
Size	3.650	3	2.468	0.065
Residuals	56.191	114		

(A) Full model statistics: adjusted *R*^2^ = 0.144, *F* = 2.314, *P* < 0.005. (B) Reduced model statistics: adj. *R*^2^ = 0.142, *F* = 2.890, *P* < 0.002. Reduced model was based on AIC. AIC, Akaike information criterion; NP, niche position; NB, niche breadth; DMG, dispersal mode group; FFG, functional feeding guild; HTG, habit trait group.

**Figure 5:**
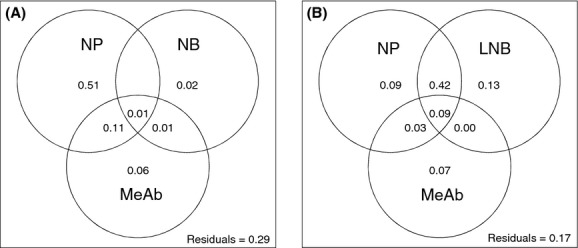
Partitioning of variation in the occupancy of species among niche position (NP), niche breadth (NB, LNB) and mean local abundance at occupied sites (MeAb). Shown are pure and shared fractions (as adjusted *R*^2^) among the explanatory variables. Subfigure (A) shows the results of niche breadth from the outlying mean index (OMI) analysis (NB), whereas subfigure (B) shows the results based on Levins' measure of niche breadth (LNB).

Variation partitioning based on linear regression showed that NP was clearly the most important variable accounting for variation in occupancy (pure effect = 51%), followed by mean local abundance (pure effect = 6%) and NB (pure effect = 2%) (Fig. 5A). It has to be emphasized that some of the variation was shared between the explanatory variables, particularly between NP and mean local abundance (shared effect = 11%). About 30% of variation in occupancy remained unexplained by these three variables. The variation partitioning results changed considerably, when the OMI-based measure of NB was replaced by Levins' measure of NB (Fig. 5B). Then, NB was the most influential variable affecting occupancy (pure effect = 13%), followed by NP (pure effect = 9%) and mean local abundance (pure effect = 7%). A high proportion of variation was shared between NP and NB (shared effect = 42%). Only 17% of variation in occupancy remained unexplained.

## Discussion

A positive relationship was found between regional occupancy and mean local abundance in stream invertebrates. Such a positive relationship is one of the most common macroecological patterns (Brown 1984; Gaston et al. 2000), and it has been found in organismal groups ranging from small-sized diatoms to large-sized mammals (Passy 2012; Slatyer et al., 2013). Previous studies in stream systems have also shown the near universality of the positive occupancy–abundance relationship, and evidence to date suggests that at least diatoms (Soininen and Heino 2005), bryophytes (Heino and Virtanen 2006), invertebrates (Heino 2005a; Siqueira et al., 2009), and fish (Tales et al. 2004) obey this pattern. However, despite positive significant relationships, the amount of explained variation around this relationship is rather low in stream systems. Such a low amount of variation explained in the occupancy–abundance relationship may be due to the fact that different species or species groups within an assemblage respond differently to environmental variation or show different spatial dynamics (Verberk et al. 2010).

Verberk et al. (2010) suggested that generalist species with broad niches should be more controlled by metapopulation dynamics, whereby high local abundance and wide regional distribution are connected by dispersal dynamics (Hanski 1982, 1994). In contrast, specialists with narrow niches should be more strongly driven by their strict requirement for suitable environmental conditions. The latter ideas pertains closely to Brown's (1984) hypothesis that broad environmental tolerances and flexible diets allow species to occur in various environments and attain high local densities. Support for both the metapopulation and NB hypotheses have previously been found in studies of stream invertebrates within a drainage basin (Heino 2005a; Heatherly et al. 2007; Siqueira et al., 2009), but little is known about the importance of these mechanisms across large spatial scales, such as across multiple drainage basins (but see Passy 2012). In the present study, the reason behind the positive occupancy–abundance relationship is unlikely to be related to metapopulation dynamics, as the three drainage basins are separated by large geographical distances, and dispersal between sites among drainage basins is unlikely to happen within short time periods. In contrast, due to large environmental gradients across the three drainage basins, niche-based mechanisms can be expected to play a stronger role in determining the positive occupancy–abundance relationship (Passy 2012). However, Brändle and Brandl (2001) suggested that, at a very large spatial extent spanning a whole continent, local habitat niches are less well associated with distribution than at smaller regional extents. Given that our study was clearly regional in spatial extent, rather than local or continental, it is perhaps not too surprising that niche characteristics were important for occupancy.

An additional question relates to the relative importance of the two niche-based hypotheses, that is, NB as environmental tolerance (Brown 1984; Slatyer et al., 2013) and NP as habitat availability (Hanski et al. 1993; Venier and Fahrig 1996). It can be assumed that both niche-based mechanisms could account for variation in occupancy and abundance and, hence, the positive occupancy–abundance relationship across large environmental gradients. However, in our study, it was evident that NP was strongly related to occupancy, whereas it accounted for only a small amount of variation in mean local abundance. By contrast, NB was only weakly related to occupancy and did not account for significant variation in mean local abundance. It is thus likely that niche characteristics, particularly NP, are important mostly for occupancy, whereas mean local abundance is related to some other factors or traits of species in stream invertebrates.

Occupancy was indeed better modeled than abundance using niche characteristics. This finding could be due to the fact that abundance is likely to vary more stochastically than occupancy, that is, the abundances of species may fluctuate much more than their presences across sites. Hirst and Jackson (2007) also found that abundance data may be more easily biased by natural and experimental error than the presence–absence data that are used for calculating occupancy. There is thus a viable hypothesis for further studies that occupancy is better predicted than abundance by niche characteristics, but there is also a viable alternative hypothesis that occupancy is simply easier to model than abundance.

Why is NP more important than NB or mean local abundance in accounting for variation in occupancy? If a species has a non-marginal niche and is able to occur in “typical” environmental conditions across a region, its distribution is likely to be wide (Hanski et al. 1993; Gregory and Gaston 2000; Tales et al. 2004). This relationship should be very likely in a region with large environmental variation (e.g., across multiple drainage basins; Tales et al. 2004). In comparison, NP was less important within a single boreal drainage basin (Heino 2005a vs. this study), whereas NB was more important within a single drainage basin compared with the situation across the three drainage basins (Heino 2005a vs. this study). Hence, NP may be less important within a single drainage basin, because niches are less recognizable as “non-marginal” and “marginal” at this smaller scale. In contrast, the weak relationship between NB and occupancy at the larger scale is probably related to the fact that both regionally common and rare species in terms of occupancy may have broad niches (i.e., a common species is always likely to be a generalist, but a rare species may also be a generalist and occur in a variety of environmental conditions although at a limited number of sites). Furthermore, the relative low independent effect of mean local abundance on occupancy may be accounted for by the fact that spatial dynamics are not acting and cannot connect occupancy and abundance through metapopulation dynamics at the large spatial extent in this study (see also Verberk et al. 2010).

The measure of NB affected this finding, though. If the variation partitioning of occupancy among the three explanatory variables, mean local abundance, NP and NB, was based on Levins' (1968) measure of NB, then NB superseded the importance of NP and mean local abundance (Fig. 4B). A likely reason behind this finding is that, based on the same data set, LNB is not mathematically independent of occupancy. The same reason could also be invoked to account for the strong effect of NP in the main analyses of variation in occupancy (Fig. 4A). However, as the OMI analysis measures species distributions along environmental gradients, it is not as strongly related, a priori, to the occupancy data as Levins' NB measure. Furthermore, if the aim is to model species environmental niches, then the OMI analysis is certainly superior to the Levins' measure (see also Devictor et al. 2010).

Biological traits were generally less important than niche characteristics in explaining variation in occupancy and abundance. This finding may be partly related to the rather coarse nature of the biological trait variables we used. However, dispersal mode explained significant variation in regional occupancy in simple analyses and in mean local abundance when other biological and ecological traits were accounted for in general linear models. Active terrestrial dispersers were more common regionally than species in the other two DMGs. An ecological reason for the former finding is likely to be that active dispersers are able to find suitable sites, where they can attain large population size. However, Statzner et al. (2008) stated that this would be unlikely and, rather, that active species should be less abundant than passive species. Again, it is possible that the different variables describing dispersal modes between studies explain some of the discrepancy, although also ecological reasons (e.g., spatial extent, regional delineations and species pools) and taxonomic issues (e.g., mostly species vs. genus level data) may be underlying the differences between these studies.

To conclude, NP was the most important variable accounting for variation in the regional occupancy of stream invertebrates. This finding is in slight contrast to Passy's (2012) suggestion that NB should attain a stronger role than NP in accounting for occupancy and abundance. Undoubtedly, both niche characteristics are important in determining the regional occupancy of species, but their relative importance may be contingent on the regional delineations, ecological settings and methods to measure species niches. Future studies should examine the effects of these factors on regional occupancy in stream invertebrates and other organismal groups.
